# Basic fibroblast growth factor regulates phosphate/pyrophosphate regulatory genes in stem cells isolated from human exfoliated deciduous teeth

**DOI:** 10.1186/s13287-018-1093-9

**Published:** 2018-12-10

**Authors:** Nunthawan Nowwarote, Waleerat Sukarawan, Prasit Pavasant, Brian L. Foster, Thanaphum Osathanon

**Affiliations:** 1 Center of Excellence for Regenerative Dentistry, Chulalongkorn University, Bangkok, 10330 Thailand; 2 Department of Pediatric Dentistry, Faculty of Dentistry, Chulalongkorn University, Bangkok, 10330 Thailand; 3 Department of Anatomy, Faculty of Dentistry, Chulalongkorn University, 34 Henri-Dunant Road, Bangkok, 10330 Thailand; 4 Division of Biosciences, College of Dentistry, The Ohio State University, Columbus, OH 43210 USA; 5 Genomics and Precision Dentistry Research Unit, Chulalongkorn University, Bangkok, 10330 Thailand

**Keywords:** Basic fibroblast growth factor, Stem cells isolated from human exfoliated deciduous teeth, Phosphate, Pyrophosphate

## Abstract

**Background:**

Basic fibroblast growth factor (bFGF) regulates maintenance of stemness and modulation of osteo/odontogenic differentiation and mineralization in stem cells from human exfoliated deciduous teeth (SHEDs). Mineralization in the bones and teeth is in part controlled by pericellular levels of inorganic phosphate (P_i_), a component of hydroxyapatite, and inorganic pyrophosphate (PP_i_), an inhibitor of mineralization. The progressive ankylosis protein (gene *ANKH*; protein ANKH) and ectonucleotide pyrophosphatase phosphodiesterase 1 (*ENPP1*/ENPP1) increase PP_i_ and inhibit mineralization, while tissue-nonspecific alkaline phosphatase (*ALPL*; TNAP) is a critical pro-mineralization enzyme that hydrolyzes PP_i_. We hypothesized that regulation by bFGF of mineralization in SHEDs occurs by modulation of P_i_/PP_i_-associated genes.

**Methods:**

Cells were isolated from human exfoliated deciduous teeth and characterized for mesenchymal stem cell characteristics. Cells were treated with bFGF, and the osteogenic differentiation ability was determined. The mRNA expression was evaluated using real-time polymerase chain reaction. The mineralization was examined using alizarin red S staining.

**Results:**

Cells isolated from primary teeth expressed mesenchymal stem cell markers, CD44, CD90, and CD105, and were able to differentiate into osteo/odontogenic and adipogenic lineages. Addition of 10 ng/ml bFGF to SHEDs during in vitro osteo/odontogenic differentiation decreased *ALPL* mRNA expression and ALP enzyme activity, increased *ANKH* mRNA, and decreased both P_i_/PP_i_ ratio and mineral deposition. Effects of bFGF on *ALPL* and *ANKH* expression were detected within 24 h. Addition of 20 mM fibroblast growth factor receptor (FGFR) inhibitor SU5402 revealed the necessity of FGFR-mediated signaling, and inclusion of 1 μg/ml cyclohexamide (CHX) implicated the necessity of protein synthesis for effects on *ALPL* and *ANKH*. Addition of exogenous 10 μm PP_i_ inhibited mineralization and increased *ANKH*, collagen type 1a1 (*COL1A1*), and osteopontin (*SPP1*) mRNA, while addition of exogenous P_i_ increased mineralization and osterix (*OSX*), *ANKH*, *SPP1*, and dentin matrix protein 1 (*DMP1*) mRNA. The effects of PP_i_ and P_i_ on mineralization could be replicated by short-term 3- and 7-day treatments, suggesting signaling effects in addition to physicochemical regulation of mineral deposition.

**Conclusion:**

This study reveals for the first time the effects of bFGF on P_i_/PP_i_ regulators in SHEDs and implicates these factors in how bFGF directs osteo/odontogenic differentiation and mineralization by these cells.

**Electronic supplementary material:**

The online version of this article (10.1186/s13287-018-1093-9) contains supplementary material, which is available to authorized users.

## Background

Basic fibroblast growth factor (bFGF; also known as FGF2) is a member of the fibroblast growth factor family of related signaling molecules crucial for development, maintenance, and repair of tissues. While it regulates various cell processes including proliferation, migration, and differentiation, bFGF also controls maintenance of stemness in human stem cells [1, 2]. In this regard, bFGF preserves the undifferentiated state of human pluripotent stem cells [3]. Downregulation of OCT3/4 is observed in human embryonic stem cells treated with fibroblast growth factor receptor (FGFR) inhibitor, suggesting a role for bFGF in regulation of genes governing pluripotency [4]. Hence, bFGF is employed as one of the key components in growth medium for human embryonic stem cells and human induced pluripotent stem cells.

Stem cells isolated from human exfoliated deciduous teeth (SHEDs) were reported in 2003 [5]. The appeal of these postnatal mesenchymal stem cells (MSCs) is that they can be obtained non-invasively (isolated after natural exfoliation of primary teeth) and show the multipotent ability to differentiate into osteogenic, adipogenic, and neurogenic lineages [6]. Although they are considered to be MSCs based on known developmental processes, SHEDs exhibit novel characteristics compared with bone marrow-derived MSCs, possibly due in part to their origin from cranial neural crest-derived ectomesenchyme and/or different patterns of signaling that occur in the craniofacial-dental region compared to the postcranial skeleton [5].

Functions of bFGF have been established in regulating some aspects of SHED biology. As it does for embryonic stem cells, addition of bFGF to culture medium maintains stemness in SHEDs in long-term culture [7]. In this context, bFGF enhances colony-forming unit (CFU) ability and the expression of pluripotent markers (e.g., *OCT4*, *REX1*, and *NANOG*) [7], but does not influence cell proliferation [7, 8]. bFGF plays a potent role in regulating the differentiation of SHEDs into an odontoblast-like phenotype. Previous reports demonstrate that bFGF attenuates tissue-nonspecific alkaline phosphatase (gene: *ALPL*; protein: TNAP) expression and mineral deposition by SHEDs and dental pulp stem cells (DPSCs), in vitro [1, 9]. bFGF inhibits *RUNX2* and *BGLAP* mRNA expression as well as mineral deposition by SHEDs in vitro via the regulation of ERK and Wnt signaling [8]. In addition, bFGF treatment of SHEDs leads to the reduction of ectopic bone formation in vivo [8]. Further, inhibition of endogeneous bFGF function using a chemical inhibitor of fibroblast growth factor receptor (FGFR) leads to increased mineralization upon osteogenic induction [6]. Correspondingly, SHEDs transfected with shRNA against bFGF exhibit higher mineral deposition than controls [6]. All of this accumulated evidence strongly supports a negative influence of bFGF on differentiation of SHEDs into mature, mineralizing odontoblast-like cells.

Inorganic phosphate (P_i_) and pyrophosphate (PP_i_) play crucial roles in physiological and pathological extracellular matrix (ECM) mineralization. P_i_ is a primary component of hydroxyapatite crystals that are deposited in the biomineralization of the bone and teeth, while PP_i_ is a potent inhibitor of crystal precipitation and growth [10]. In addition to their physicochemical roles in mineralization, both P_i_ and PP_i_ have been reported to have signaling effects on cells, though mechanisms remain incompletely understood. The addition of P_i_ promoted mineralization in rat osteoblasts [11]. Conversely, PP_i_ supplementation resulted in reduction of mineral deposition in vitro with reduced cell proliferation and collagen synthesis in murine cementoblasts [12]. Local pericellular P_i_ and PP_i_ concentrations are regulated by TNAP, ectonucleotide pyrophosphatase/phosphodiesterase-1 (*ENPP1*;ENPP1), and the progressive ankylosis protein (*ANKH*; ANKH in humans and *Ank*/ANK in mice) [10]. TNAP cleaves extracellular PP_i_ into P_i_, facilitating ECM mineralization. ENPP1 increases extracellular PP_i_ by cleaving nucleotide triphosphates, while ANKH regulates transport of intracellular PP_i_ to the extracellular space. Functions of ANKH and ENPP1 result in increased extracellular PP_i_ and subsequently inhibit mineralization. Studies on human loss-of-function mutations and genetically engineered mouse models have demonstrated that TNAP, ENPP1, and ANKH/ANK have profound effects on skeletal and dental mineralization [12–18].

To date, little is known about how bFGF affects expression of P_i_/PP_i_ regulators. Studies using MC3T3.E1 murine pre-osteoblasts showed that bFGF upregulates *Enpp1* and *Ank* mRNA expression, while *Alpl* mRNA levels are downregulated [19]. Another report demonstrated that bFGF inhibits *ALPL* expression in SHEDs [9]. However, an influence of bFGF on the other key P_i_/PP_i_ regulatory genes in SHEDs has not yet been investigated, making it unclear how these genes contribute to bFGF regulation of osteo/odontoblast differentiation and mineralization. In the present study, we aimed to investigate in SHEDs the effect of bFGF on P_i_ and PP_i_ regulatory genes and roles of P_i_ and PP_i_ on mineralization of SHEDs.

## Methods

### Cell isolation and culture

The study was approved by Human Research Ethics Committee, Faculty of Dentistry, Chulalongkorn University (Approval number 2015-007). The procedure was performed according to the Declaration of Helsinki. Informed consent was obtained from parents. Primary teeth with no pathological lesions scheduled for extraction according to the clinical treatment plan were collected and stored in culture medium. Dental pulp tissues were obtained, and an explantation protocol was applied for cell isolation, using 35-mm tissue culture plate [2, 6]. The migrated cells were subcultured when cell confluence was achieved. Cells were cultured in Dulbecco’s modified Eagle medium (DMEM, Gibco, USA) supplemented with 10% fetal bovine serum (Gibco, USA), 2 mM l-glutamine (Gibco, USA), 100 U/mL penicillin (Gibco, USA), 100 μg/mL streptomycin (Gibco, USA), and 5 μg/mL amphotericin B (Gibco, USA). Cells were maintained in 100% humidity at 37 °C and 5% carbon dioxide.

In experiments described below, cells were treated with the following reagents: 10 ng/ml recombinant human bFGF (Invitrogen, USA), 20 mM FGFR inhibitor (SU5402; Calbiochem, USA), 5 mM sodium phosphate (Na_2_HPO_4_; Sigma-Aldrich, USA), 10 uM sodium pyrophosphate tetrabasic (Na_4_O_7_P_2_; Sigma-Aldrich, USA), and 1 μg/ml cyclohexamide (CHX; Sigma-Aldrich, USA). ALP was generously provided by Assistant Professor Jeerus Sucharitakul (Faculty of Dentistry, Chulalongkorn University, Thailand).

### Flow cytometry

Single cell suspensions were obtained by detaching cells with trypsin/EDTA solution. Cells were centrifuged, and the supernatant culture medium was discarded. Cells were rinsed with 1% FBS in PBS and further immunostained with primary antibodies conjugated to fluorescent dye, including anti-human CD44 (BD Bioscience Pharmingen, USA), PerCP-CyTM5.5-conjugated anti-human CD90 (BD Bioscience Pharmingen, USA), PE-conjugated anti-human CD105 (BD Bioscience Pharmingen, USA), and PerCP-conjugated anti-CD45 (BD Bioscience Pharmingen, USA). Cells were analyzed using a FACSCalibur flow cytometer using CellQuest software for operation and gating (BD Bioscience, USA).

### Osteo/odontogenic induction

Cells were seeded in 24-well plates at a density of 50,000 cells per well. After 24 h, culture medium was replaced with osteogenic induction medium consisting of growth medium supplemented with 252.39 μM L(+)-ascorbic acid sodium salt, 10 mM β-glycerophosphate, and 100 nM dexamethasone. The medium was changed every 48 h. Mineral deposition was analyzed using alizarin red S staining, as described below. Osteogenic marker gene expression was determined using quantitative real-time polymerase chain reaction, as described below. To test involvement of FGF signaling, in some experiments, SHEDs were pretreated with an FGFR inhibitor (SU5402) 30 min prior to bFGF exposure and cells were maintained in osteogenic induction medium supplemented with bFGF and SU5402 for the length of the experiment.

### Adipogenic induction

Cells were seeded in 24-well plates at a density of 12,500 cells per well. Adipogenic induction medium consisted of growth medium supplemented with 0.1 mg/mL insulin, 1 mM dexamethasone, 1 mM isobutylmethylxanthine (IBMX), and 0.2 mM indomethacin. The medium was changed every 72 h. Oil Red O staining was performed to evaluate intracellular lipid accumulation according to a previously published protocol [2].

### Alkaline phosphatase activity (ALP) assay

Cells were lysed in an alkaline lysis buffer. The samples were then incubated with *p-*nitrophenol phosphate solution (2 mg/mL, Invitrogen, USA). After 15 min, the reaction was stopped by the addition of 50 mM NaOH. The absorbance was measured at 410 nm. Total protein was evaluated using a bicinchoninic acid (BCA) assay (Thermo Scientific, USA). ALP activity was calculated and normalized to the amount of total protein.

### Alizarin red S staining

Alizarin red S staining was employed to determine calcium deposition in culture [20]. Cells were fixed with cold methanol for 10 min and washed with deionized water. Subsequently, cells were incubated with 1% alizarin red S solution for 3 min at room temperature under gentle agitation. Cells were then washed with deionized water to remove excess staining. Precipitated dye was solubilized in 10% *w*/*v* cetylpyridinium chloride solution, and the absorbance was measured at 570 nm.

### Quantitative polymerase chain reaction (QPCR)

RNA isolation was performed using RiboEx total RNA isolation solution (GeneAll, Seoul, South Korea). Complimentary DNA (cDNA) was synthesized from RNA using reverse transcriptase ImPromII kit (Promega, Madison, WI, USA). QPCR was performed using FastStart Essential DNA Green Master kit (Roche Diagnostic, USA) on the MiniOpticon real-time PCR system (Bio-Rad, USA). Target gene expression values were normalized to *18S* expression values and further normalized to controls. Oligonucleotide sequences are listed in Additional file 1: Table S1.

### Phosphate and pyrophosphate assay

After osteo/odontogenic differentiation, the culture medium was collected and analyzed using the EnzChek® Phosphate assay kit (Molecular PROBES, Oregon, USA) for quantitative analysis of P_i_ and the EnzChek® Pyrophosphate assay kit (Molecular PROBES, Oregon, USA) for detecting free PP_i_ following the manufacturer’s protocol. Absorbance was measured at 360 nm.

### Statistical analyses

Each experiment was performed using cells from at least four different donors. The Mann-Whitney *U* test was employed for comparisons of two groups. For comparison of three or more groups, the Kruskal-Wallis test was performed, followed by pairwise comparison with type I error correction. All statistical analyses were performed using Prism 7 (GraphPad Software, CA, USA). Statistical significance was indicated by *p* < 0.05.

## Results

### SHED cell isolation and characterization

**Fig. 1:**
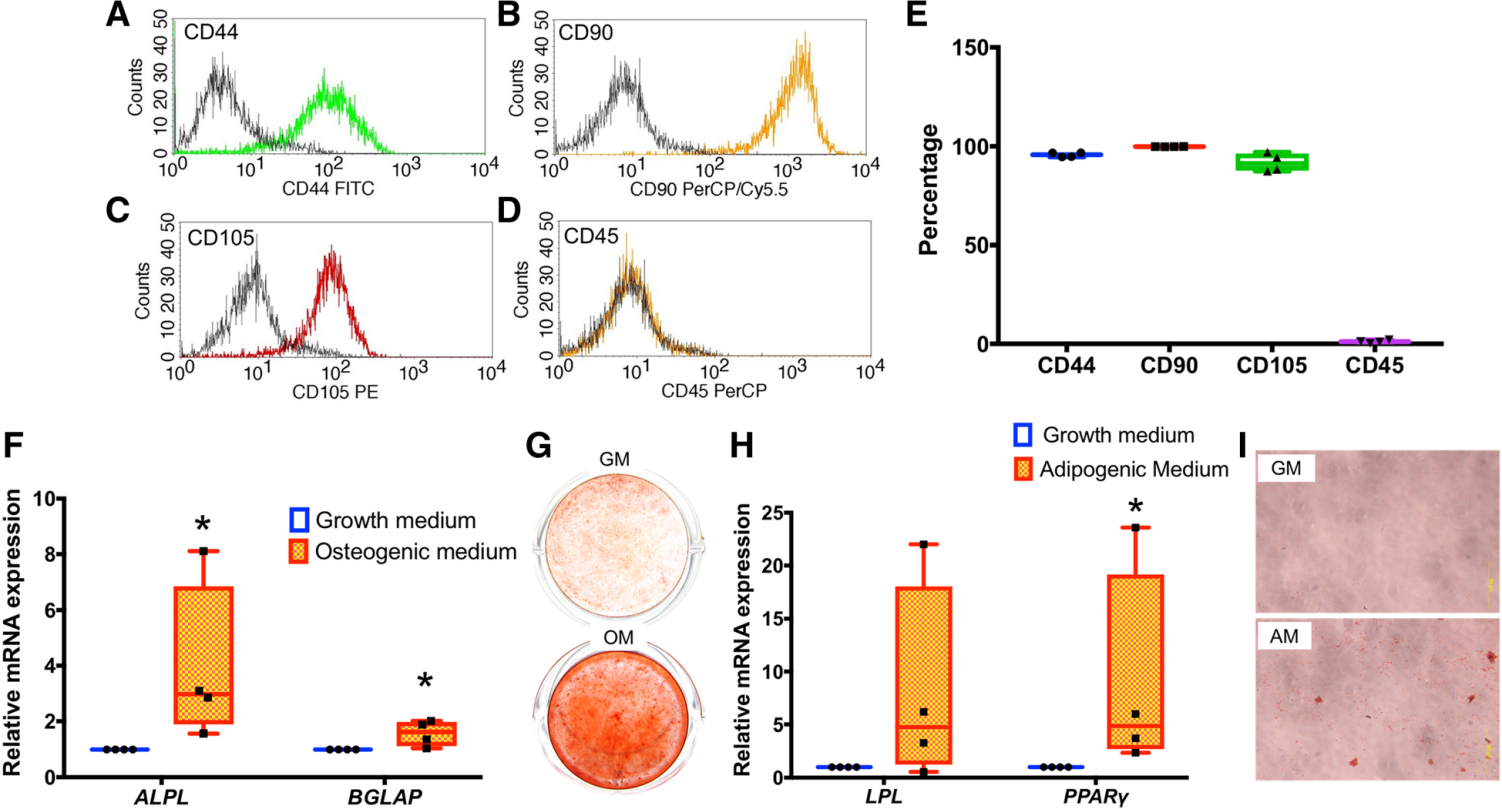
SHED cell isolation and characterization. Cells harvested from primary teeth were analyzed by flow cytometry and found to express MSC markers, **a** CD44, **b** CD90, and **c** CD105, but lack expression of hematopoietic stem cell marker, **d** CD45. **e** MSC markers are expressed in more than 90% of cells, with CD45 expressed in only about 1% of cells. **f** Significant upregulation of *ALPL* and *BGLAP* mRNA expression is observed after osteogenic induction of SHED cells for 7 days. **g** Increased mineral deposition by SHED cells under osteogenic induction is evident after 14 days. **h** Upregulation of *LPL* and *PPARγ* is observed after adipogenic induction of SHED cells for 8 days. **i** Intracellular lipid accumulation is observed after adipogenic induction for 16 days. GM growth medium, OM osteogenic induction medium, AM adipogenic induction medium. Asterisks indicated the statistically significant difference compared to the control (*p* < 0.05)

Cells were harvested from pulp chambers of the extracted primary teeth. Expression of mesenchymal and hematopoietic stem cell surface markers was evaluated using flow cytometry. Consistent with expectations for SHEDs, the isolated cells expressed MSC markers, CD44, CD90, and CD105 (Fig. 1a–c), but lacked expression of hematopoietic stem cell marker, CD45 (Fig. 1d). The percentage of cells expressing CD44, CD90, and CD105 was over 95%, 99%, and 92%, respectively (Fig. 1e). In contrast, only 1% were CD45-expressing cells. Significant upregulation of *ALPL* and bone γ-carboxyglutamate protein (*BGLAP*) mRNA expression was observed after osteo/odontogenic induction for 7 days (Fig. 1f), and after 14 days, a marked increase of mineral deposition was evident (Fig. 1g). Upregulation of adipogenic markers, lipoprotein lipase (*LPL*) and peroxisome proliferator-activated receptor *γ* (*PPARγ*), was observed after adipogenic induction for 8 days (Fig. 1h), and intracellular lipid accumulation was subsequently observed after 16 days (Fig. 1i). This combined evidence of MSC markers and multipotent differentiation ability confirms the MSC-like characteristics of the isolated SHED cells.

### bFGF inhibits in vitro mineralization and alters osteo/odontogenic gene expression in SHEDs

**Fig. 2:**
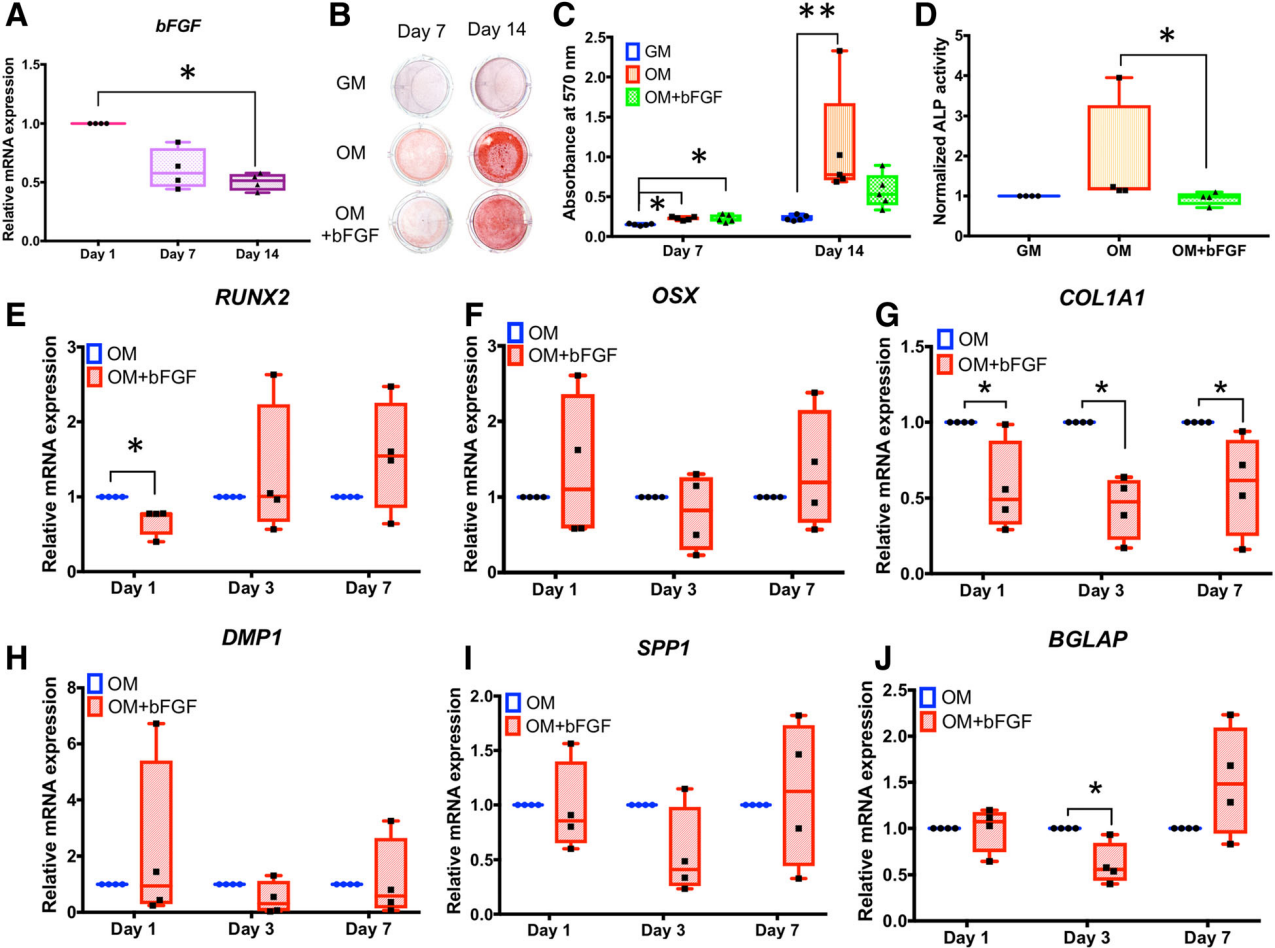
bFGF inhibits in vitro mineralization and alters osteogenic/odontogenic gene expression in SHEDs. **a** Expression of *bFGF* is reduced 50% (*p* < 0.05) in SHED cells during osteogenic induction over 14 days. Inclusion of 10 ng/mL bFGF in osteogenic induction medium significantly attenuates mineral deposition at 14 days (**b**, **c**) and reduces ALP enzymatic activity at 7 days (**d**). **e**–**j** Addition of bFGF reduces mRNA expression of *RUNX2* (day 1), *COL1A1* (days 1, 3, and 7), and *BGLAP* (day 3) (*p* < 0.05 for all), but does not significantly affect expression of *OSX*, *SPP1*, or *DMP1*. The Mann-Whitney *U* test was employed for comparisons of two groups. The Kruskal-Wallis test followed by pairwise comparison was used for comparison of three groups. Bars indicated the statistically significant difference (*p < 0.05, **p < 0.01). GM growth medium, OM osteogenic induction medium

During osteo/odontogenic induction over 14 days, *bFGF* mRNA levels were reduced in a time-dependent manner until they were decreased by about 50% (*p* < 0.05) (Fig. 2a). Addition of 10 ng/mL bFGF to odontogenic induction medium led to the significant attenuation of mineral deposition at 14 days (*p* < 0.01) (Fig. 2b, c). This observation correlated with reduced ALP enzymatic activity in the bFGF-treated group after osteo/odontogenic induction for 7 days (Fig. 2d). Based on effects of bFGF on in vitro mineralization and ALP, we further investigated effects on genes associated with osteo/odontogenic differentiation and mineralization (Fig. 2e–j). bFGF treatment reduced mRNA expression of transcription factor, Runt-related transcription factor 2 (*RUNX2*), on day 1 (40%; *p* < 0.05), but did not affect *OSX* expression (Fig. 2e, f). bFGF decreased *COL1A1* mRNA (by 40–50%; *p* < 0.05) on all days (Fig. 2g) and *BGLAP* (by 40%; *p* < 0.05) on day 3 (Fig. 2h), but did not affect expression of *SPP1* or *DMP1* (Fig. 2i, j).

### bFGF alters expression of phosphate/pyrophosphate regulatory genes in SHEDs

**Fig. 3:**
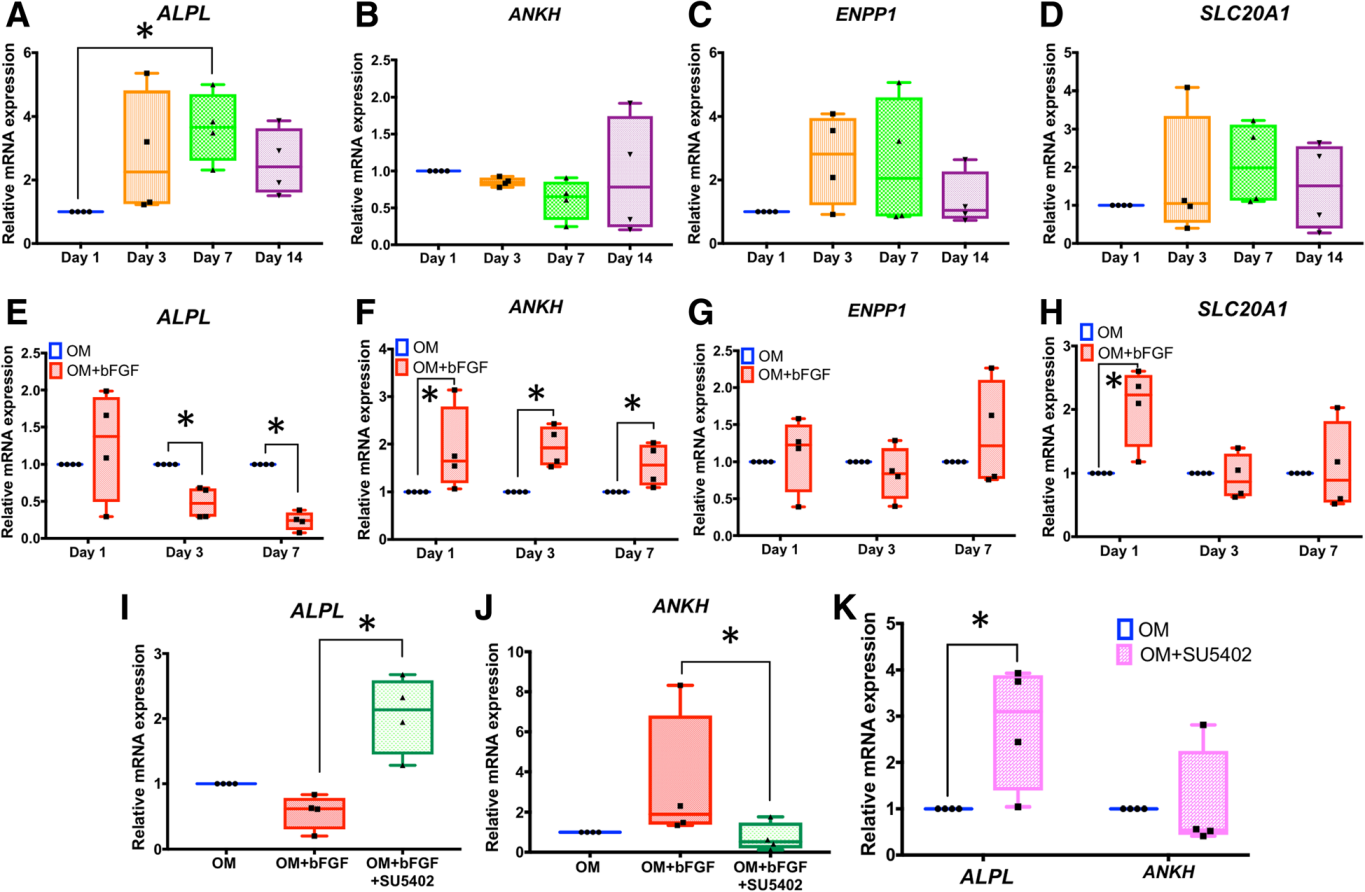
bFGF alters expression of phosphate/pyrophosphate regulatory genes in SHEDs. **a** SHED cells cultured in osteogenic medium over 14 days increase *ALPL* mRNA (day 7; *p* < 0.05), with no significant alterations in **b** *ANKH*, **c** *ENPP1*, or **d** *SLC20A1* expression. Inclusion of 10 ng/mL bFGF in osteogenic induction medium **e** reduces *ALPL* mRNA (days 3 and 7), **f** increases *ANKH* mRNA (days 1, 3, and 7), **g** does not significantly affect *ENPP1* mRNA, and **h** increases *SLC20A1* mRNA (day 1). Addition of 20 mM FGFR inhibitor (SU5402) inhibits bFGF-induced changes in **i** *ALPL* and **j** *ANKH* on day 7. **k** Addition of 20 mM SU5402 in the absence of exogenous bFGF increases *ALPL* (*p* < 0.05) but does not affect *ANKH* expression. The Mann-Whitney *U* test was employed for comparison of two groups. The Kruskal-Wallis test followed by pairwise comparison was used for comparison of three or more groups. Bars indicated the statistically significant difference. OM osteogenic induction medium (*p < 0.05)

Experiments above confirmed bFGF regulates mineralization, ALP, and osteo/odontogenic genes in SHEDs. We next sought to determine if bFGF regulates expression of P_i_/PP_i_ regulatory genes, including *ALPL*, *ANKH*, *ENPP1*, and the sodium-P_i_ transporter, solute carrier family 20 member 1 (*SLC20A1*), during osteo/odontogenic differentiation in SHEDs. Examination of mRNA levels over 14 days of osteo/odontogenic differentiation demonstrated that *ALPL* was upregulated at day 7 (3-fold; *p* < 0.05) (Fig. 3a), while expression of *ANKH* tended towards decrease and *ENPP1* and *SLC20A1* tended towards increase, though these changes were not significant within the parameters of these experiments (Fig. 3b–d).

The influence of bFGF on P_i_/PP_i_ regulatory gene expression was determined in osteogenic induction medium. bFGF treatment significantly inhibited *ALPL* mRNA expression on days 3 and 7 after (by 50% or more; *p* < 0.05) (Fig. 3e). Significant upregulation of *ANKH* mRNA was observed on all days (between 50 and 100% increased; *p* < 0.05) (Fig. 3f). bFGF did not alter *ENPP1* expression during osteo/odontogenic induction (Fig. 3g) and induced *SLC20A1* mRNA (more than 100%; *p* < 0.05) on day 1 but not on later days (Fig. 3h).

SHEDs were pretreated with an FGFR inhibitor (SU5402) to confirm the involvement of bFGF signaling in modulation of *ALPL* and *ANKH* mRNA. SU5402 supplementation attenuated the influence of bFGF on both *ALPL* and *ANKH* mRNA expression, blocking the inhibitory effect of bFGF on *ALPL* (Fig. 3i) and preventing induction of *ANKH* upregulation (Fig. 3j). Inhibition of endogenous bFGF function by addition of SU5402 to osteogenic medium (in the absence of exogenous bFGF addition) resulted in a significant increase of *ALPL* expression at 7 days (3-fold; *p* < 0.050), but no significant regulation of *ANKH* mRNA levels (Fig. 3k).

**Fig. 4:**
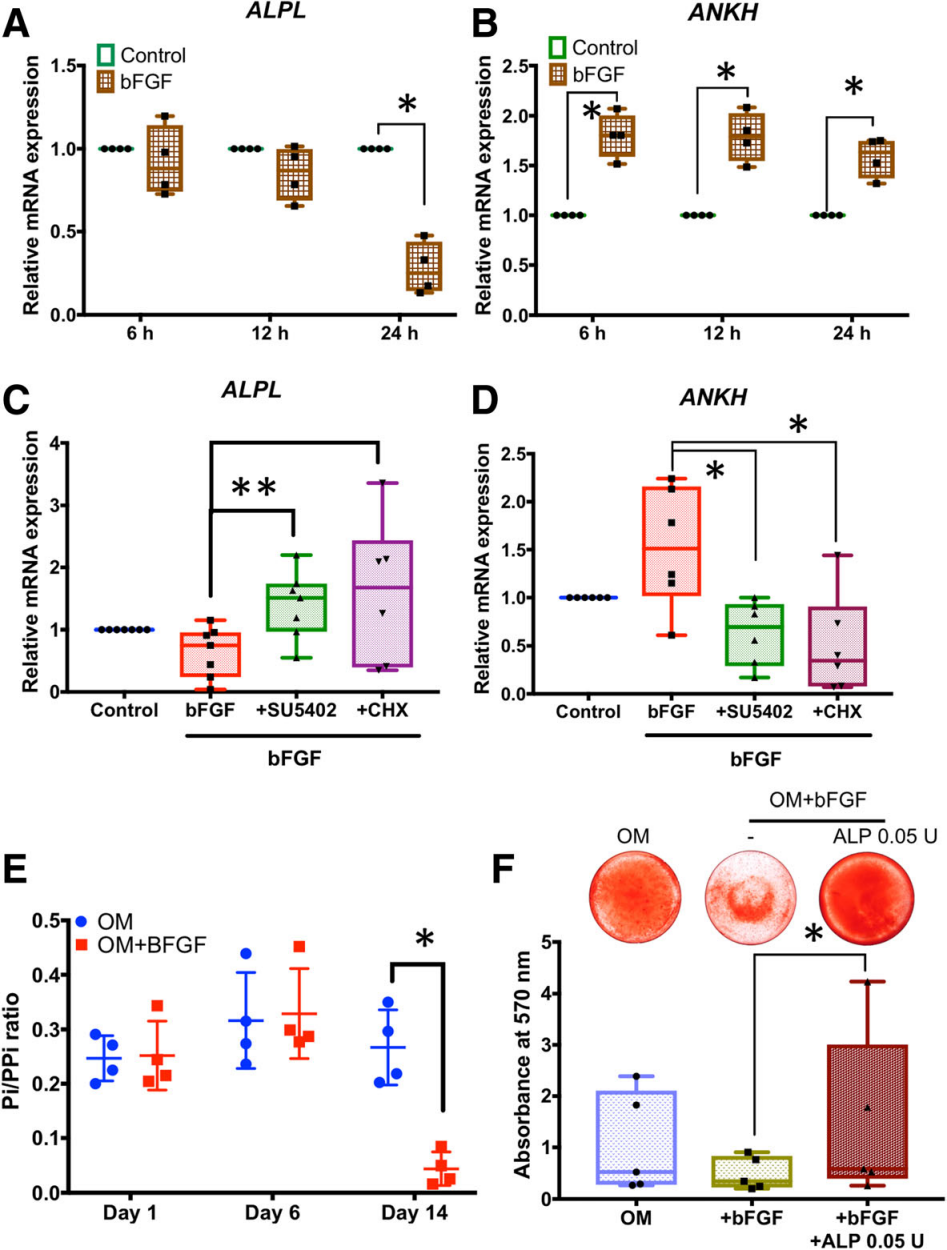
Regulation of *ALPL* and *ANKH* expression by bFGF in SHEDs. Cells were maintained in normal growth medium in the presence or absence of 10 ng/mL bFGF for 6, 12, or 24 h. **a** Addition of bFGF significantly inhibits *ALPL* mRNA by 24 h. **b** The induction of *ANKH* mRNA by bFGF is observed at 6, 12, and 24 h. **c**, **d** For both *ALPL* and *ANKH*, inclusion of 20 mM FGFR inhibitor (SU5402) in growth medium abolishes effects of bFGF at 24 h. Addition of 1 μg/ml cyclohexamide (CHX) inhibits effects of bFGF on *ALPL* and *ANKH* expression, suggesting that protein translation is required. **e** In culture medium collected from SHEDs on days 1, 6, and 14 after osteogenic induction, bFGF significantly reduces P_i_/PP_i_ ratio by day 14. **f** Addition of 0.05 U ALP rescues bFGF-attenuated *ALP* expression and mineral deposition in SHEDs. The Mann-Whitney *U* test was employed for comparisons of two groups. The Kruskal-Wallis test followed by pairwise comparison was used for comparisons of three or more groups. Bars indicate statistically significant differences (*p < 0.05, **p < 0.01). OM osteogenic induction medium

In time course experiments, cells were supplemented with bFGF and maintained in growth medium with expression assayed at 6, 12, and 24 h. bFGF inhibition of *ALPL* mRNA began between 12 and 24 h (about 70% decrease; *p* < 0.05) (Fig. 4a). In contrast, the induction of *ANKH* mRNA by bFGF was observed at 6, 12, and 24 h (approximately 50–75% increase; *p* < 0.05) (Fig. 4b). Inclusion of FGFR inhibitor, SU5402, in growth medium for 24 h effectively abolished effects of bFGF on *ALPL* and *ANKH* expression (Fig. 4c, d). Inclusion of cycloheximide (CHX) in the media over 24 h also abolished effects of bFGF, strongly suggesting that protein translation is required as an intermediate step for the effects of bFGF on *ALPL* and *ANKH* (Fig. 4c, d).

Culture medium collected on days 1, 6, and 14 after osteo/odontogenic induction revealed bFGF significantly reduced P_i_/PP_i_ ratio by day 14 (by about 80%; *p* < 0.05) (Fig. 4e), confirming a functional effect from reduced *ALPL* and increased *ANKH* expression. Addition of 0.05 U ALP rescued bFGF-attenuated mineral deposition (Fig. 4f).

### Inorganic phosphate and pyrophosphate regulate mineralization by SHEDs

**Fig. 5:**
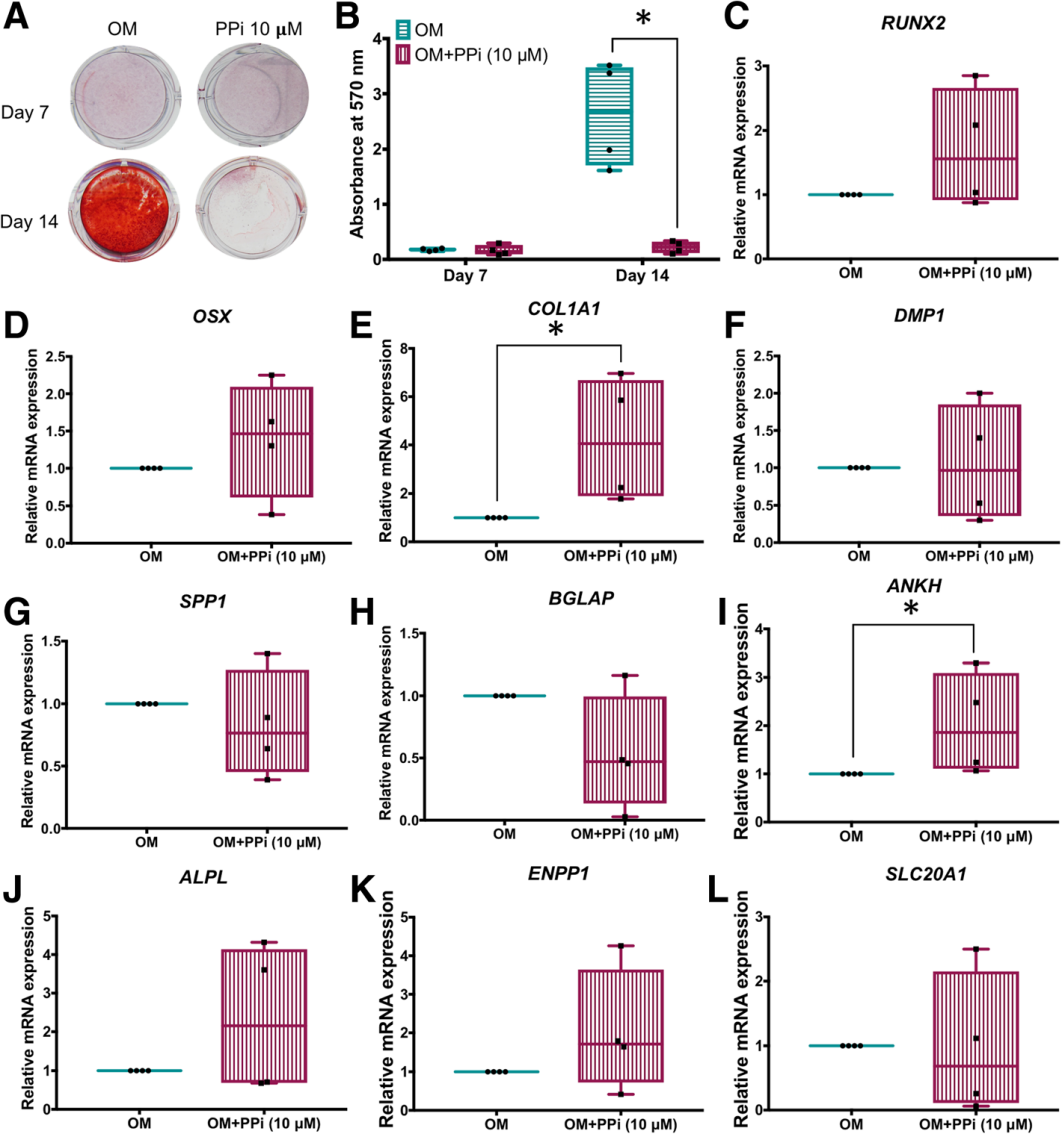
PP_i_ regulates mineralization and gene expression in SHEDs. **a**, **b** Addition of 10 μM PP_i_ to osteogenic induction medium of SHED cells nearly completely inhibits mineral deposition by 14 days. **c**–**l** At 7 days, inclusion of PP_i_ increases *COL1A1* (*p* < 0.05) and *ANKH* (*p* < 0.05), but does not significantly affect other genes associated with mineralization and P_i_/PP_i_ regulation, compared to untreated controls. The Mann-Whitney *U* test was employed for comparisons of two groups, and bars indicated the statistically significant difference (*p < 0.05). OM osteogenic induction medium

Effects of exogenous PP_i_ on osteoblast and cementoblast mineralization in vitro have been reported [12, 21]; however, to our knowledge, PP_i_ has not been tested on stem cells, or more specifically on SHEDs. Addition of 10 μM PP_i_ to osteogenic induction medium of SHEDs nearly completely inhibited mineral deposition by 14 days (Fig. 5a, b). At 7 days, inclusion of PP_i_ increased *COL1A1* (4-fold; *p* < 0.05) and *ANKH* (about 2-fold; *p* < 0.05), but did not significantly affect a number of other genes associated with mineralization and P_i_/PP_i_ regulation, compared to untreated controls (Fig. 5c–l).

**Fig. 6:**
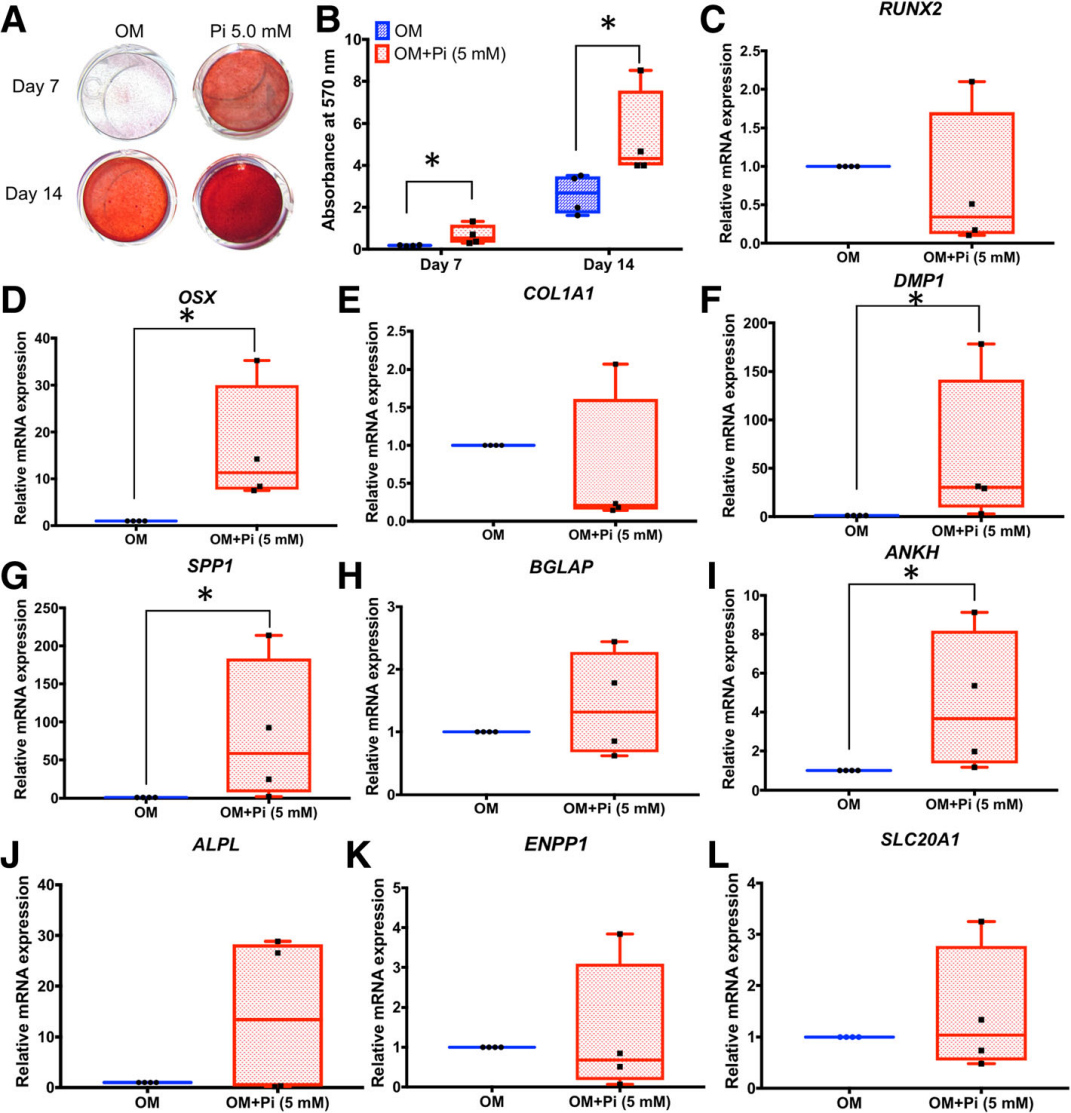
P_i_ regulates mineralization and gene expression in SHEDs. **a**, **b** Addition of 5 mM P_i_ to osteogenic induction medium of SHED cells increases mineral deposition at 7 and 14 days. **c**–**l** At 7 days, inclusion of P_i_ increases *OSX*, *DMP1*, *SPP1*, and *ANKH* (*p* < 0.05 for all), but does not significantly affect other genes associated with mineralization and P_i_/PP_i_ regulation, compared to untreated controls. The Mann-Whitney *U* test was employed for comparisons of two groups, and bars indicated the statistically significant difference (*p < 0.05). OM osteogenic induction medium

Effects of exogenous P_i_ on osteoblast, cementoblast, and odontoblast mineralization and expression in vitro have been extensively studied [22–25], and experiments investigating effects of P_i_ on various types of stem cells have been reported [26, 27]. However, to our knowledge, this relationship has not been explored in SHEDs or other dental stem cells. Addition of 5 mM P_i_ to osteogenic medium significantly increased mineral deposition by SHEDs at both days 7 and 14 (about 3- and 2-fold, respectively; *p* < 0.05 for both) (Fig. 6a, b). At 7 days, inclusion of P_i_ significantly induced *OSX* (10-fold), *DMP1* (25-fold), *SPP1* (50-fold), and *ANKH* (4-fold) mRNA (*p* < 0.05 for all); however, other genes were unaffected (Fig. 6c–l).

**Fig. 7:**
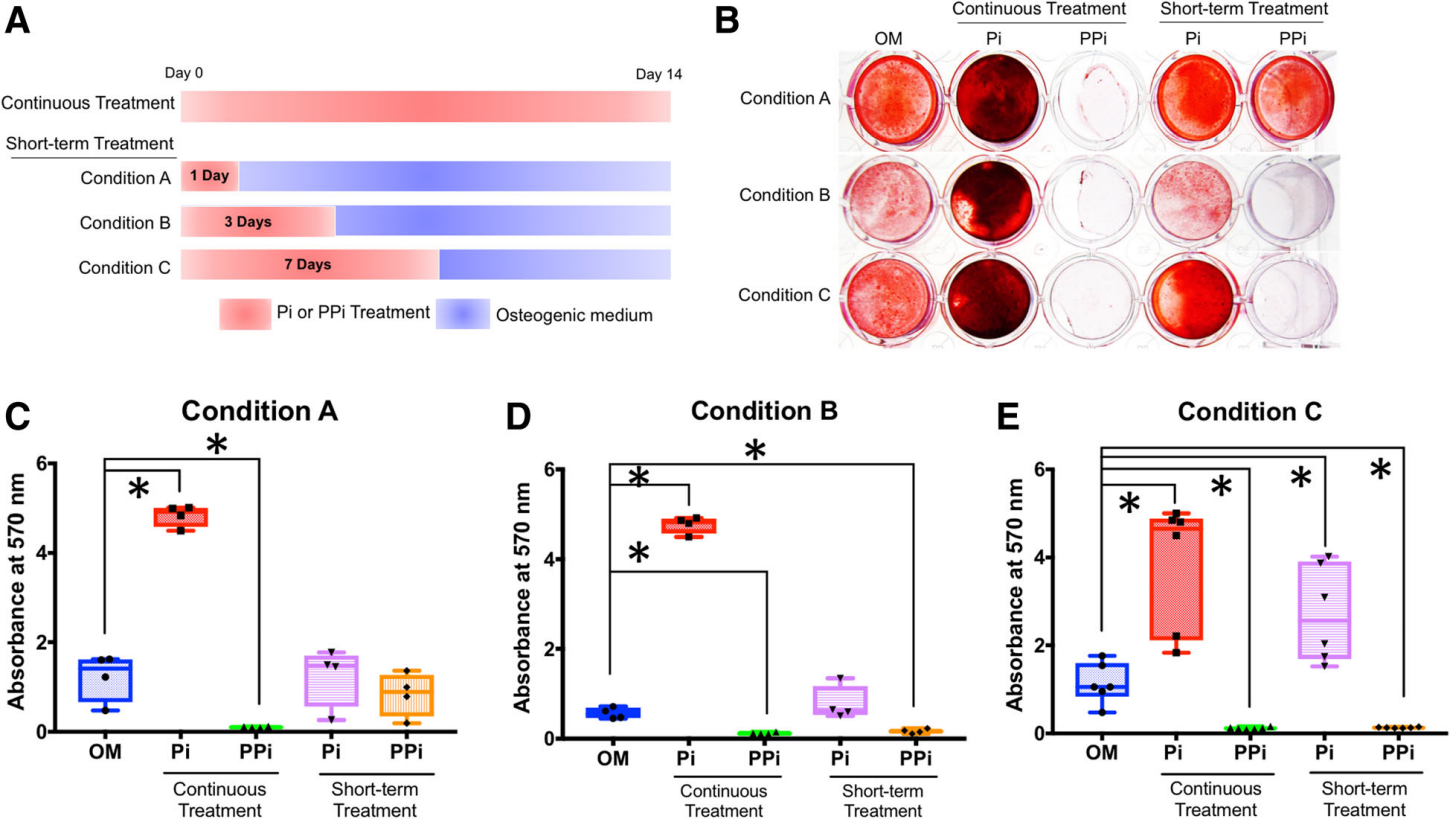
Regulation of mineralization in SHEDs by continuous and short-term P_i_ and PP_i_ treatment. **a** Cells were treated with 5 mM P_i_ or 10 μM PP_i_ in osteogenic medium, with no P_i_ or PP_i_ serving as negative control, and continuous administration of P_i_ or PP_i_ serving as the positive control, whereas conditions A, B, and C incorporated shorter-term P_i_ or PP_i_ treatments of 1, 3, or 7 days, respectively, followed by completion of the experiment in osteogenic medium. **b** Continuous treatment of SHEDs with P_i_ significantly increases (*p* < 0.05) mineral deposition while or continuous PP_i_ significantly decreases (*p* < 0.05) mineral deposition. **c** Cells under condition A show no significant differences in mineralization from negative controls. **d** Cells under condition B exhibit PP_i_-mediated inhibition of mineralization by 3 days (*p* < 0.05). **e** Cells under condition C show increased mineralization from P_i_ treatment (*p* < 0.05) and decreased mineralization from PP_i_ treatment (*p* < 0.05). The Mann-Whitney *U* test was employed for comparisons of three or more groups, and bars indicated the statistically significant difference (*p < 0.05). OM osteogenic induction medium

From the above experiments, it was not clear whether P_i_ or PP_i_ was acting early or late to influence cell mineralization in the 14-day experiment. Therefore, the effect of duration of P_i_ or PP_i_ treatment was investigated. Continuous administration of 5 mM P_i_ or 10 μM PP_i_ in osteogenic medium served as the positive control, no added P_i_ or PP_i_ served as negative control, and conditions A, B, and C incorporated shorter-term P_i_ or PP_i_ treatments of 1, 3, or 7 days, respectively, followed by completion of the experiment in osteogenic medium (Fig. 7a). Continuous treatment of SHEDs with P_i_ or PP_i_ resulted in significantly increased (nearly 3-fold; *p* < 0.05) or decreased (20-fold; *p* < 0.05) mineral deposition, respectively (Fig. 7b–e). Cells under condition A showed no significant difference in mineralization (Fig. 7b). Cells under condition B exhibited PP_i_-mediated inhibition of mineralization by 3 days (Fig. 7c). Lastly, cells under condition C showed increased mineralization from P_i_ treatment (more than 2-fold; *p* < 0.05), while those receiving PP_i_ treatment significantly reduced mineral deposition (Fig. 7d). These results within the first 7 days suggest that the supplementation of exogenous P_i_ and PP_i_ not only influences the precipitation and growth of mineral crystals, but may also affect intracellular mechanism(s).

## Discussion

For the first time, we report effects of bFGF on regulation of P_i_/PP_i_ and mineralization in SHEDs. Addition of bFGF to SHEDs during in vitro osteo/odontogenic differentiation decreased *ALPL* mRNA expression and ALP enzyme activity, increased *ANKH* mRNA, and decreased P_i_/PP_i_ ratio and mineral deposition. Effects of bFGF on *ALPL* and *ANKH* mRNA expression were detected within 24 h and required FGFR signaling and protein synthesis. Addition of exogenous PP_i_ inhibited mineralization and increased *ANKH*, *COL1A1*, and *SPP1* mRNA, while addition of exogenous P_i_ increased mineralization and increased *OSX*, *ANKH*, *SPP1*, and *DMP1* mRNA. These effects of PP_i_ and P_i_ on mineralization could be replicated by short-term 3- and 7-day treatments, suggesting early signaling effects in addition to physical-chemical modulation of mineral deposition. This study reveals the effects of bFGF on P_i_/PP_i_ regulators in SHEDs and implicates these factors in how bFGF directs osteo/odontogenic differentiation and mineralization by these cells.

### Regulation by bFGF of osteo/odontogenic markers and mineralization in SHED cells

Previous studies demonstrated that bFGF inhibited mineralization in SHEDs and DPSCs [1, 9]. Here, we confirmed this effect of bFGF on mineralization in SHEDs and extended understanding of the underlying mechanisms by examining the influence of bFGF on other osteo/odontogenic markers and P_i_/PP_i_ regulators. Type 1 collagen, encoded by *COL1A1* and *COL1A2* genes, composes 90% of the extracellular matrix (ECM) of the bone, dentin, and cementum. bFGF significantly inhibited *COL1A1* expression during osteo/odontogenic differentiation on days 1, 3, and 7. It has been shown that bFGF suppresses *COL1* and *COL3* while enhancing mRNA expression of matrix metalloproteinases *MMP1* and *MMP2* in human periodontal ligament (PDL) cells, suggesting a catabolic influence of bFGF on ECM regulation [28]. Similarly, collagen synthesis was reduced by bFGF treatment in rat hair dermal papilla cells [29]. Hence, the bFGF-induced reduction of *COL1A1* expression could be beneficial in terms of scar formation during wound healing. On the contrary, in rabbit chondrocytes, bFGF did not influence *COL1A1* mRNA levels but suppressed *COL2* mRNA expression [30]. Tenocytes overexpressing bFGF increased *COL1* and *COL3* expression.

We demonstrated that bFGF inhibits *RUNX2* and *BGLAP* mRNA expression on days 1 and 3, respectively. RUNX2 serves a master transcription factor regulating osteo/odontogenic differentiation and function, upstream of numerous key genes [31, 32]. *BGLAP* encodes OCN, an extracellular matrix protein found in the bone, dentin, and cementum and posited to regulate mineralization as well as perform wider endocrine functions [33, 34]. Previous publications implicated bFGF inhibition of osteo/odontogenic differentiation of SHEDs in part via reduction of *RUNX2* and *BGLAP* mRNA [8]. Similarly, studies on stem cells from apical papilla (SCAP) showed that bFGF treatment during osteo/odontogenic induction decreased *BGLAP* mRNA levels at early but not late time points [35]. In PDL cells, bFGF inhibited mineralization when added to osteogenic medium, but with no apparent regulation of *RUNX2* or *BGLAP* expression, suggesting alternative mechanisms [36]. However, bFGF induced *RUNX2* expression in human periodontal ligament cells which are maintained in growth medium [36]. In mouse osteoblasts, bFGF attenuated RUNX2-induced *Alpl* expression [37]. The influence of bFGF on *RUNX2* and *OCN* at specific time points during osteo/odontogenic induction of SHEDs and other cells requires further investigation.

These collected data concerning effects of bFGF on *COL1A1*, *RUNX2*, and *BGLAP* suggest cell-type-specific responses to bFGF. The reduction of all three of these osteo/odontogenic markers concomitant with decreased mineral deposition supports a link to attenuation of mineralization; however, additional studies are warranted to explore these relationships further.

### Effect of bFGF on P_i_ and PP_i_ regulators in SHED cells

During osteo/odontogenic differentiation, SHEDs upregulated *ALPL* expression early, while *ANKH*, *ENPP1*, and *SLC20A1* mRNA levels were not markedly changed during the differentiation period assayed here. *Alpl* was induced early in OCCM.30 murine cementoblasts, and *Ank*, *Enpp1*, *Spp1*, *and Dmp1* mRNA increased at later times after mineral deposition [38]. In MC3T3.E1 murine pre-osteoblasts, bFGF induced *Enpp1* and *Ank* but inhibited *Alpl* mRNA expression [19]. In MLO-Y4 murine osteocyte-like cells, bFGF upregulated *Ank*, *Enpp1*, *Slc20a1*, and *Dmp1* [39]. Conversely, we showed here that bFGF suppressed *ALPL* but induced *ANKH* expression in SHEDs, while *ENPP1* expression was not markedly changed by exposure to bFGF. These changes were blocked by addition of an FGFR inhibitor, confirming the requirement of FGF signaling. In addition, the effect of bFGF on *ALPL* and *ANKH* expression was attenuated by protein synthesis inhibitor, suggesting the involvement of intermediate factors in the regulation process. Ultimately, the consequence of bFGF-reduced *ALPL* and increased *ANKH* in SHEDs was borne out by significantly decreased P_i_/PP_i_ ratio, an alteration that likely contributed to reduced mineralization in vitro and would be anticipated to have a potent effect to inhibit ECM mineralization.

Based on inhibition of mineralization and changes in P_i_/PP_i_ regulatory genes in response to bFGF, we explored direct effects of exogenous PP_i_ or P_i_ on SHEDs during osteo/odontogenic differentiation. Addition of exogenous PP_i_ inhibited mineralization and significantly upregulated *COL1A1* and *ANKH* expression in SHEDs. In OCCM.30 cementoblasts, 10–100 μM PP_i_ decreased mineral deposition, and removal of PP_i_ from culture medium during early stages allowed mineral deposition to proceed as normal, suggesting a primarily physicochemical effect on mineral deposition by these cells [12]. In contrast, a dose of 2.5 μM PP_i_ increased *Alpl* and *Ank* and decreased *Enpp1* in MC3T3.E1 pre-osteoblasts, implicating direct signaling effects [21]. We documented that withdrawal of PP_i_ from medium after 1 day allowed SHEDs to mineralize similar to untreated controls; however, PP_i_ withdrawal after days 3 or 7 did not reverse the inhibitory effect of PP_i_ on mineralization. Because SHEDs largely mineralize between 7 and 14 days in osteogenic medium, these results suggest that PP_i_ not only inhibits mineral precipitation and growth, but may also regulate cells through additional signaling mechanisms, a hypothesis that requires further investigation. In *Alpl* knockout (*Alpl*^*−/−*^) mice, a model for the hereditary error-in-metabolism, hypophosphatasia (HPP), loss of TNAP function increases PP_i_ and prevents mineralization in the bones and teeth. Severely hypomineralized dentin in *Alpl*^*−/−*^ mice was accompanied by loss of expression of *Bglap* and dentin sialophosphoprotein (*Dspp*) mRNA by odontoblasts [40], supporting a connection between PP_i_, mineralization, and altered expression in skeletal/dental cells in vivo.

Addition of 5 mM P_i_ significantly increased *OSX*, *DMP1*, *SPP1*, and *ANKH* mRNA in SHEDs. P_i_ is well established as a potent signal in osteoblasts, cementoblasts, and odontoblasts [22–25, 41]. *Spp1*/OPN was first identified as a target for P_i_ signaling in MC3T3.E1 murine pre-osteoblasts, where extracellular signal-regulated kinases (ERK) 1/2 and protein kinase C (PKC) pathways were implicated in its transcription [25, 41]. Subsequently, P_i_ was found to regulate numerous genes in osteoblasts [41–43]. In OCCM.30 cementoblasts, P_i_ also induced *Spp1*/OPN, possibly through a glucocorticoid receptor in the promoter region [22, 44]. OPN is a multifunctional ECM protein involved in cell migration, attachment, differentiation, and also a negative regulator of mineralization proposed to be involved in pathological hypomineralization of dentin in diseases such as HPP and X-linked hypophosphatemia (XLH) [40, 45–47]. Genetic ablation of OPN in *Spp1*^*−/−*^ mice resulted in increased mineral content in the bones, increased cortical and trabecular bone parameters in femurs and tibias, and increased dentin and alveolar bone [48–50]. Interestingly, *Spp1*^*−/−*^ mice featured defective reparative dentin formation, suggesting the protein is necessary for pulpo-dentinal healing [51].

DMP1 is an ECM protein closely related to OPN that is expressed by odontoblasts, osteoblasts, and osteocytes. P_i_ induction of *Dmp1*/DMP1 was previously documented in OCCM.30 murine cementoblasts and human PDL fibroblasts [12, 52]. DMP1 is critical for dentin formation and periodontal development. *Dmp* knockout (*Dmp1*^*−/−*^) mice exhibit a dentinogenesis imperfecta-like phenotype including thin and hypomineralized dentin and an enlarged pulp cavity in the molars [53]. Additionally, loss-of-function of DMP1 is responsible for autosomal recessive hypophosphatemic rickets, an endocrine disorder marked by low circulating P_i_ and severe hypomineralization of the bones and teeth [54].

OSX is a transcription factor directing osteoblast and odontoblast differentiation [55, 56]. The upregulation of *OSX* in SHEDs by P_i_ observed in the present study paralleled P_i_ induced *Osx* expression in rat kidney fibroblast and human vascular smooth muscle cells [57, 58]. In those cells, P_i_ induced upregulation of *Osx* occurred via Akt1 and ERK signaling [57].

Addition of P_i_ to SHEDs increased expression of *ANKH* mRNA. Induction of *Ank* was also achieved by addition of 5 mM P_i_ to OCCM.30 murine cementoblast cells, hypothesized to be a response to pro-mineralization conditions and a sort of negative feedback to compensate the overabundance of extracellular P_i_ and to balance P_i_/PP_i_ ratio, and regulate the pace of mineralization [22]. Loss of function of *Ank/ANKH* leads to decreased PP_i_ and increased and sometimes ectopic mineralization, and *Ank* mutant or knockout mice feature skeletal differences and massively increased acellular cementum [12, 14, 17, 59]. These potent effects of ANK/ANKH on mineralized tissue development in vivo justify further investigation of the interactions of bFGF, *ANKH*, PP_i_, and mineralization in SHEDs.

### Influence of local P_i_ and PP_i_ regulation on tooth development and regeneration

P_i_ and PP_i_ participate in tooth development and regeneration. Their roles were demonstrated by the investigation in knockout and mutated mouse models. *Alpl* mutation, resulting in increased local PP_i_ concentrations, causes delayed tooth eruption, aplasia or hypoplasia of cementum, and defective insertion of Sharpey’s fibers [10, 60]. Conversely, mice lacking *Ank* or *Enpp1* function exhibited hypercementosis with otherwise normal dental and periodontal structure [10, 61]. These data strongly support the significant role of local P_i_/PP_i_ ratio via regulation by TNAP, ANK/ANKH, and ENPP1, in directing tooth development and structural formation.

Besides the role of P_i_/PP_i_ in tooth development, modulation of this ratio has also been proposed as an alternative approach for regenerative treatment. TNAP immobilized fibrin scaffolds enhanced P_i_ concentration and osteogenic differentiation in vitro as well as increased calvarial bone regeneration in vivo [62]. Local delivery of polyphosphate led to the increase alveolar bone regeneration around rat molars [63]. Studies of *Ank* knockout mice demonstrated that decreased PP_i_ promoted more rapid and greater cementum regeneration in a periodontal defect model compared to wild-type mice [64], implying a role for P_i_/PP_i_ regulation in periodontal tissue regeneration. However, more work must indeed be done to understand the mechanism(s) and to develop treatment strategies in translational and clinical settings.

### Benefit of bFGF in future clinical applications

The present study aimed to investigate regulatory mechanisms whereby bFGF affected SHED differentiation and mineralization. We concluded that continuous bFGF treatment of SHEDs inhibited mineral deposition, in part via regulation of genes controlling P_i_/PP_i_ metabolism. However, several reports demonstrated that bFGF pretreatment before osteogenic induction promoted osteogenic potential of various human stem/progenitor cells [65]. Our findings parallel those of Fakhry and colleagues, which indicated that continuous treatment of calvarial osteoblasts with bFGF inhibited osteogenic gene expression and mineralization, while short-term treatment of bFGF stimulated osteogenic differentiation [66]. Moreover, previous publications reported the beneficial utilization of bFGF in hard tissue formation through various approaches. Local application of bFGF enhanced dental implant stability and osseointegration in animal models [67, 68]. In addition, bFGF has been utilized in alveolar bone and periodontal tissue regeneration, i.e., periodontal defect treatment and alveolar ridge preservation [69, 70]. The anabolic effect of bFGF in vivo could result from the brief exposure of local cells to the released proteins, prompting osteogenic differentiation. In addition, bFGF regulates angiogenesis, cell migration, and cell proliferation [71, 72]. Hence, these processes could help to facilitate the healing and formation of hard tissue in vivo*.*

Another potential use of bFGF in clinical regenerative therapy would be to maintain stemness of stem and progenitor populations in vitro*.* It has been shown that mesenchymal stem cells isolated from dental tissues lose their stem cell properties and decline in proliferation during long-term culture in vitro [7]*.* Our previous reports revealed that continuous bFGF treatment in vitro promoted maintenance of stemness in dental tissue-derived mesenchymal stem cells [1, 2, 7]. The bFGF supplementation in long-term culture of SHEDs enhanced colony-forming unit ability and stem cell marker gene expression [7]. Hence, bFGF could be useful to amplify cell number while maintaining stem cell properties in vitro for future regenerative application of SHEDs.

## Conclusions

This study reveals the effects of bFGF on osteogenic differentiation ability of SHEDs. In addition, it has been demonstrated that bFGF influences P_i_/PP_i_ regulators in SHEDs and implicates these factors in how bFGF directs osteo/odontogenic differentiation and mineralization by these cells.

## Additional file

Additional file 1: **Table S1.** Primer sequences for QPCR. (DOCX 18 kb)

## Abbreviations

Akt: Serine/threonine-specific protein kinase
ALP: Alkaline phosphatase
ALPL: Tissue-nonspecific alkaline phosphatase
ANKH: Progressive ankylosis protein
BCA: Bicinchoninic acid
bFGF: Basic fibroblast growth factor
BGLAP: Bone γ-carboxyglutamate protein
CD105: Cluster of differentiation 105
CD44: Cluster of differentiation 44
CD45: Cluster of differentiation 45
CD90: Cluster of differentiation 90
cDNA: Complimentary DNA
CHX: Cycloheximide
COL1: Collagen type 1
COL12: Collagen type 2
COL1A1: Collagen type 1a1
COL1A2: Collagen type 1a2
COL3: Collagen type 3
DMP1: Dentin matrix protein 1
DPSCs: Dental pulp stem cells
Dspp: Dentin sialophosphoprotein
ECM: Extracellular matrix
ENPP1: Ectonucleotide pyrophosphatase phosphodiesterase 1
ERK: Extracellular signal-regulated kinases
FGFR: Fibroblast growth factor receptor
HPP: Hypophosphatasia
IBMX: Isobutylmethylxanthine
LPL: Lipoprotein lipase
MMP: Matrix metalloproteinases
MSCs: Mesenchymal stem cells
NANOG: Nanog homeobox
NaOH: Sodium hydroxide
OCCM.30: Murine cementoblast cell line
OCN: Osteocalcin
OCT4: POU class 5 homeobox 1
OPN: Osteopontin
OSX: Osterix
PDL: Periodontal ligament
P_i_: Inorganic phosphate
PKC: Protein kinase C
PPARγ: Peroxisome proliferator-activated receptor *γ*
PP_i_: Inorganic pyrophosphate
REX1: ZFP42 zinc finger protein
RUNX2: Runt-related transcription factor 2
SCAP: Stem cells from apical papilla
SHEDs: Stem cells isolated from human exfoliated deciduous teeth
SLC20A1: Solute carrier family 20 member 1
SPP1: Secreted phosphoprotein 1
SU5402: FGFR inhibitor
TNAP: Tissue-nonspecific alkaline phosphatase
XLH: X-linked hypophosphatemia
