# Supplementary material for: Basic fibroblast growth factor regulates phosphate/pyrophosphate regulatory genes in stem cells isolated from human exfoliated deciduous teeth
Source: Stem Cell Res Ther. 2018 Dec 10;9:345. doi: 10.1186/s13287-018-1093-9 (PMC6288970; doi:10.1186/s13287-018-1093-9)
Supplement: Supplementary file 1 — Table S1. Primer sequences for QPCR. (DOCX 18 kb) [file 13287_2018_1093_MOESM1_ESM.docx]

**Table S1.** Primer sequences.

**Gene Accession Number Primer sequences**

*bFGF* NM_002006.4 (Forward) 5’ GGCTTCTTCCTGCGCATCCAC 3’

(Reverse) 5’ TAACGGTTAGCACACACTC CTT 3’

*ALPL*  NM000478.5 (Forward) 5’ GACCTCCTCGGAAGACACTC 3’

(Reverse) 5’ TGAAGGGCTTCTTGTCTGTG 3’

*COL1A1* NM000088.3 (Forward) 5’ GTGCTAAAGGTGCCAATGGT 3’

(Reverse) 5’ ACCAGGTTCACCGCTGTTAC 3’

*DMP1* NM 004407.3 (Forward) 5’ CAGGAGCACAGGAAAAGGAG 3’

(Reverse) 5’ CTGGTGGTATCTTGGGCACT 3’

*RUNX2* NM001024630.3 (Forward) 5’ ATGATGACACTGCCACCTCTG 3’

(Reverse) 5’ GGCTGGATAGTGCATTCGTG 3’

*OSX* NM001173467.2 (Forward) 5' GCCAGAAGCTGTGAAACCTC 3'

(Reverse) 5’ GCTGCAAGCTCTCCATAA 3’

*LPL*  NM000237.2 (Forward) 5’ GAGATTTCTCTGTATGGCACC 3'

(Reverse) 5' CTGCAAATGAGACACTTTCTC 3'

*PPARγ*  NM138712.3 (Forward) 5′CCAGTGGTTGCAGATTACAAGTATG 3′

(Reverse) 5′TTGTAGAGCTGAGTCTTCTCAGAATAATAAG 3'

*BGLAP* NM199173.5 (Forward) 5’ CTTTGTGTCCAAGCAGGAGG 3’

(Reverse) 5’ CTGAAAGCCGATGTGGTCAG 3’

*SPP1*  NM001040058.1 (Forward) 5’ AGGAGGAGGCAGAGCACA 3’

(Reverse) 5’ CTGGTATGGCACAGGTGATG 3’

*ANKH*  NM054027.4 (Forward) 5’ GAGGTGACAGACATCGTGG 3'

(Reverse) 5' CCTTTAAATCAAGGCCTCTTTCATTAC 3'

*SCL20A1* NM005415.4 (Forward) 5’ GGAGGGTGTCAAGTGGTCTGAA 3’

(Reverse) 5’ ATCTGCCTTATGGAGGATGAATG 3’

*ENPP1* NM006208.2 (Forward) 5’ AAATATGCAAGCCCTCTTTGT 3’

(Reverse) 5’ TTTAGAAGGTGGTTAAGACTTCCATGA 3’

*18S* NR003286.2 (Forward) 5’ GGCGTCCCCCAACTTCTTA3’

(Reverse) 5’ GGGCATCACAGACCTGTTATT 3’
